# Optimization of artificial urine formula for *in vitro* cellular study compared with native urine

**DOI:** 10.7150/ijms.61720

**Published:** 2021-07-23

**Authors:** Kanyarat Sueksakit, Visith Thongboonkerd

**Affiliations:** Medical Proteomics Unit, Office for Research and Development, Faculty of Medicine Siriraj Hospital, Mahidol University, Bangkok 10700, Thailand

**Keywords:** AU-Siriraj, Cell polarization, Fetal bovine serum, Protein supplement, Renal tubular cell, Transepithelial electrical resistance

## Abstract

Several artificial urine (AU) formulas have been developed to mimic the normal urine. Most of them are protein-free, particularly when secreted proteins (secretome) is to be analyzed. However, the normal urine actually contains a tiny amount of proteins. We hypothesized that urinary proteins at physiologic level play a role in preservation of renal cell biology and function. This study evaluated the effects from supplementation of 0-10% fetal bovine serum (FBS) into the well-established AU-Siriraj protocol on MDCK renal tubular cells. Time to deformation (T_D_) was reduced by both native urine and AU-Siriraj without/with FBS compared with complete culture medium (control). Among the native urine and AU-Siriraj without/with FBS, the cells in AU-Siriraj+2.5% FBS had the longest T_D_. Supplementation of FBS increased cell death in a dose-dependent manner (but still <10%). Transepithelial electrical resistance (TER) of the polarized cells in the native urine was comparable to the control, whereas that of the cells in AU-Siriraj+2.5% FBS had the highest TER. These data indicate that supplementation of 2.5% FBS into AU-Siriraj can prolong time to deformation and enhance polarization of renal tubular cells. Therefore, AU-Siriraj+2.5% FBS is highly recommended for *in vitro* study of cell biology and function (when secretome is not subjected to analysis).

## 1. Introduction

Artificial urine (AU) has been developed to mimic the native human urine for various *in vitro* studies. Several AU formulas have been reported using different kinds of chemicals with various ranges of their concentrations 1. One of those is our previously established formula, AU-Siriraj, which has been proven as one of the most physiologic AU formulas compared with the other protocols 1. As such, the AU-Siriraj formula has been widely used in several recent studies for disease mechanisms 2, diagnostics 3, biomarker discovery 4, drug delivery 5-9, crystallization and crystal growth 10, biosensors/bioelectronics 11, 12, etc. Moreover, a recent study has shown that AU-Siriraj can improve the polarization features of renal tubular epithelial cells using a Transwell system 13. Most of the AU formulas are protein-free to address the effects of treatments, interventions, or experimental inductions on secretory proteins (or secretome) 1. Note that exogenous proteins can definitely interfere with the analysis and interpretation of changes in the secretion of endogenous proteins 14-16.

The native urine contains mainly water and several various small molecules, including urea, sodium, potassium, chloride, creatinine, phosphate and other compounds 17, 18. In addition, the native urine from human and other mammals contains a tiny amount of macromolecules, particularly proteins 19. One among these urinary proteins is albumin, of which the high level is an important indicator for several kidney diseases, such as diabetic nephropathy, hypertension and chronic kidney disease (CKD) 19, 20. The normal or physiologic level of urinary albumin is < 30 mg/24 h (or mg/g creatinine) (normoalbuminuria). Moderately and severely increased albumin levels are considered when urinary albumin levels are 30-300 mg/24 h (or mg/g creatinine) (microalbuminuria) and > 300 mg/24 h (or mg/g creatinine) (macroalbuminuria), respectively 19. In addition to albumin, the normal urine contains several other proteins, e.g., immunoglobulins, transferrin, α1-antitrypsin serine proteinase inhibitors, kininogen and bikunin 21, 22. Normal to mildly increased, moderately increased, and severely increased proteinuria are classified when urinary protein levels are < 150, 150-500, and > 500 mg/24 h (or mg/g creatinine), respectively 19.

We therefore hypothesized that the normal or physiologic urinary proteins play a role in preservation of renal tubular cell biology and function. In the normal urine, approximately 70% of urinary proteins are derived from glomerular filtration of the serum/plasma proteins, whereas the rest (30%) are from renal tubular secretion (in free forms or as the urinary exosomes or extracellular vesicles) 23-25. This study thus aimed to evaluate the effects from supplementation of various concentrations of proteins from fetal bovine serum (FBS) into the well-established AU-Siriraj protocol on floating, monolayered and polarized Madin-Darby Canine Kidney (MDCK) renal tubular epithelial cells.

## 2. Materials and Methods

### 2.1. Human subjects and urine collection

All the experiments involving human subjects and clinical samples were reviewed and approved by the Siriraj Institutional Review Board (approval no. Si650/2015). The experiments involving clinical samples were conducted according to the international guidelines, i.e., the Declaration of Helsinki, the Belmont Report, and ICH Good Clinical Practice. Late afternoon midstream urine samples were collected from 12 healthy individuals (2 males and 10 females, aged 31.5 ± 1.18 years). The urine samples were centrifuged at 300 ×*g* and 4°C for 10 min to eliminate cellular debris. An equal volume of urine samples (10 ml each) from four individuals were randomly combined as one pool to make three different pools for all subsequent experiments.

### 2.2. Preparation of AU-Siriraj without or with exogenous protein supplementation

AU-Siriraj was prepared according to the protocol described previously 1, 13. The final compositions of this AU were 200 mM urea, 1 mM uric acid, 4 mM creatinine, 5 mM Na_3_C_6_H_5_O_7_·2H_2_O, 54 mM NaCl, 30 mM KCl, 15 mM NH_4_Cl, 3 mM CaCl_2_·2H_2_O, 2 mM MgSO_4_·7H_2_O, 2 mM NaHCO_3_, 0.1 mM NaC_2_O_4_, 9 mM Na_2_SO_4_, 3.6 mM NaH_2_PO_4_·H_2_O, and 0.4 mM Na_2_HPO_4_. Final pH, specific gravity, and osmolality of this physiologic AU were 6.2, 1.010 (g/ml), and 446 (mOsm/kg), respectively. To examine the effects of exogenous protein supplementation, 0.625%, 1.25%, 2.5%, 5% or 10% fetal bovine serum (FBS) **(**Gibco, Invitrogen; Grand Island, NY**)** was added into the AU-Siriraj formula.

### 2.3. Cell culture

**Figure 1:**
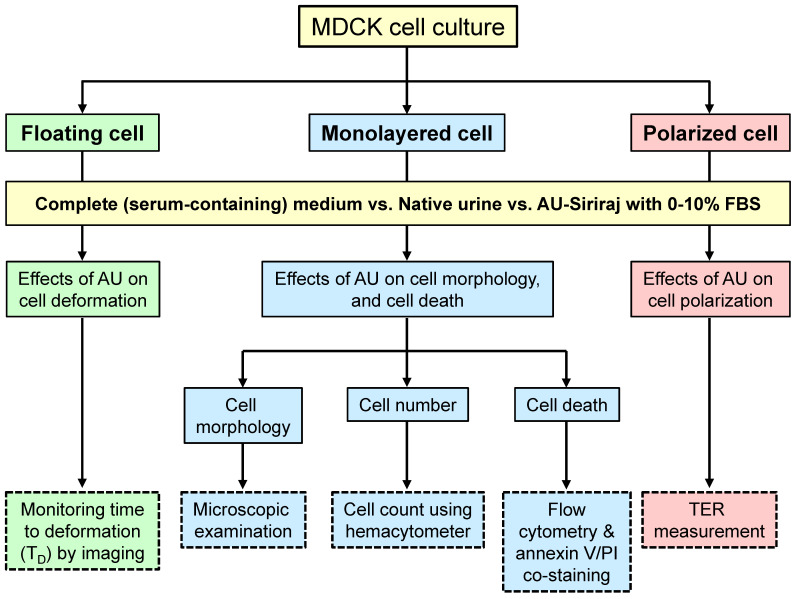
** Schematic summary of the study design.** Effects of supplementation of fetal bovine serum (FBS) to the artificial urine (AU) on floating, monolayered and polarized MDCK renal tubular epithelial cells were evaluated. Such supplementation was compared with the complete (serum-containing) medium (control) and three different pools of the native urine. Time to deformation (T_D_), cell morphology, cell number, cell death, and transepithelial electrical resistance (TER) were examined.

MDCK cell line (strain II) (ATCC; Manassas, VA), the most commonly used cell line/strain for *in vitro* epithelial cell polarity studies 26, was employed. These MDCK cells were maintained in a complete culture medium **(**containing Eagle**'**s minimum essential medium** (**MEM**) (**Gibco**)** supplemented with 10**%** (v/v) heat-inactivated FBS (Gibco), 60 U/ml penicillin G (Sigma, St. Louis; MO), and 60 µg**/**ml streptomycin (Sigma)) in a humidified incubator containing 5**%** CO_2_ at 37°C**.** The medium was refreshed every day. Floating, monolayered and polarized cells were then prepared for subsequent experiments as follows (see also **Figure 1**).

### 2.4. Analysis of time to deformation (T_D_) in the floating cells

The floating cells were used for evaluation of cell tolerance to the AU or native urine. The cells (approximately 7.5×10^4^ cells) were seeded in each well of the 24-well plate (Corning Costar; Cambridge, MA) with 1 ml of the complete (serum-containing) culture medium. After incubation in a humidified incubator containing 5**%** CO_2_ at 37°C for 24 h, the cells were washed with PBS and trypsinized with 0.1% trypsin in 2.5 mM EDTA/PBS. Thereafter, the cell suspension was centrifuged at 300 ×*g* and 4°C for 3 min and the supernatant containing trypsin was removed. The cells were then resuspended in 1 ml of the complete (serum-containing) medium, native urine, or AU-Siriraj with 0-10% FBS supplement. Cell morphology was monitored every 10 min under the Nikon Eclipse Ti-S inverted phase-contrast light microscope (Nikon; Tokyo, Japan). Time to deformation (T_D_) was recorded for each sample.

### 2.5. Analysis of cell morphology, total number and death in the monolayered cells

MDCK cells (approximately 1.0×10^5^ cells) were seeded in each well of the 6-well plate (Corning Costar) and incubated in complete (serum-containing) medium for 48 h to allow the monolayer to develop. The cells were incubated in a humidified incubator containing 5**%** CO_2_ at 37°C and the medium was refreshed every day. Thereafter, the monolayer was washed with PBS and the cells were further incubated with the complete (serum-containing) medium, native urine, or AU-Siriraj with 0-10% FBS supplement for 1 h. Cell morphology was imaged using the Nikon Eclipse Ti-S inverted phase-contrast light microscope. The adherent cells were then collected by trypsinization using 0.1% trypsin in 2.5 mM EDTA/PBS. The total cell number was counted using a hemacytometer.

In parallel, the trypsinized cells were washed with ice-cold PBS. Thereafter, the cell suspension was centrifuged at 500 ×*g* and 4ºC for 5 min. After PBS removal, the cells were resuspended with annexin V binding buffer (10 mM HEPES, 140 mM NaCl, and 2.5 mM CaCl_2_.2H_2_O, pH 7.4) (BD Biosciences; San Jose, CA) followed by incubation with FITC-conjugated annexin V (BD Biosciences) at 25°C in the dark for 15 min. Propidium iodide (BD Biosciences) was added and the cell suspension was further incubated for 5 min before analysis by using BD Accuri C6 flow cytometer (BD Biosciences).

### 2.6. Measurement of transepithelial electrical resistance (TER) in the polarized cells

The cells (approximately 7.5×10^4^ cells) were seeded on each collagen-coated polyethylene culture insert (1.12-cm^2^ area) of the 12-mm Transwell plate (0.4-µm pore size) (Corning Costar) and grown with the complete (serum-containing) culture medium in both upper and lower chambers of the Transwell. The cells were incubated in a humidified incubator containing 5**%** CO_2_ at 37°C and the medium was refreshed every day. After 60-h incubation, when TER no longer increased and could be stabilized 13, the medium in upper chamber was then replaced with fresh complete (serum-containing) medium, native urine or AU-Siriraj without or with 0.625%, 1.25%, 2.5%, 5% or 10% FBS supplement. After switching culture medium in the upper chamber for 24 h (or at 84-h post-culture), the polarized MDCK cells were subjected to TER measurement at three different sites in each sample well using Millicell-ERS resistance system (Millipore; Bedford, MA) 27, 28. The resistance value obtained from the sample well was then subtracted with the background obtained from the blank coated-well without cells filled with the same conditioned medium. TER values were calculated using a following equation.

TER (Ohm·cm^2^)

= (Resistance of sample well - Resistance of blank well) (Ohm) × Well area (cm^2^)

### 2.7. Statistical analysis

All quantitative data were obtained from three independent experiments and are reported as mean ± SEM unless stated otherwise. Comparisons among groups of the samples were performed using ANOVA with Tukey's post-hoc test. *P* values less than 0.05 were considered statistically significant.

## 3. Results

This study examined the effects from supplementation of various concentrations (0-10%) of FBS into the well-established AU-Siriraj protocol on floating, monolayered and polarized MDCK renal tubular cells. The cells in a complete culture medium supplemented with 10% FBS served as the control. The study design is summarized in **Figure 1**.

**Figure 2:**
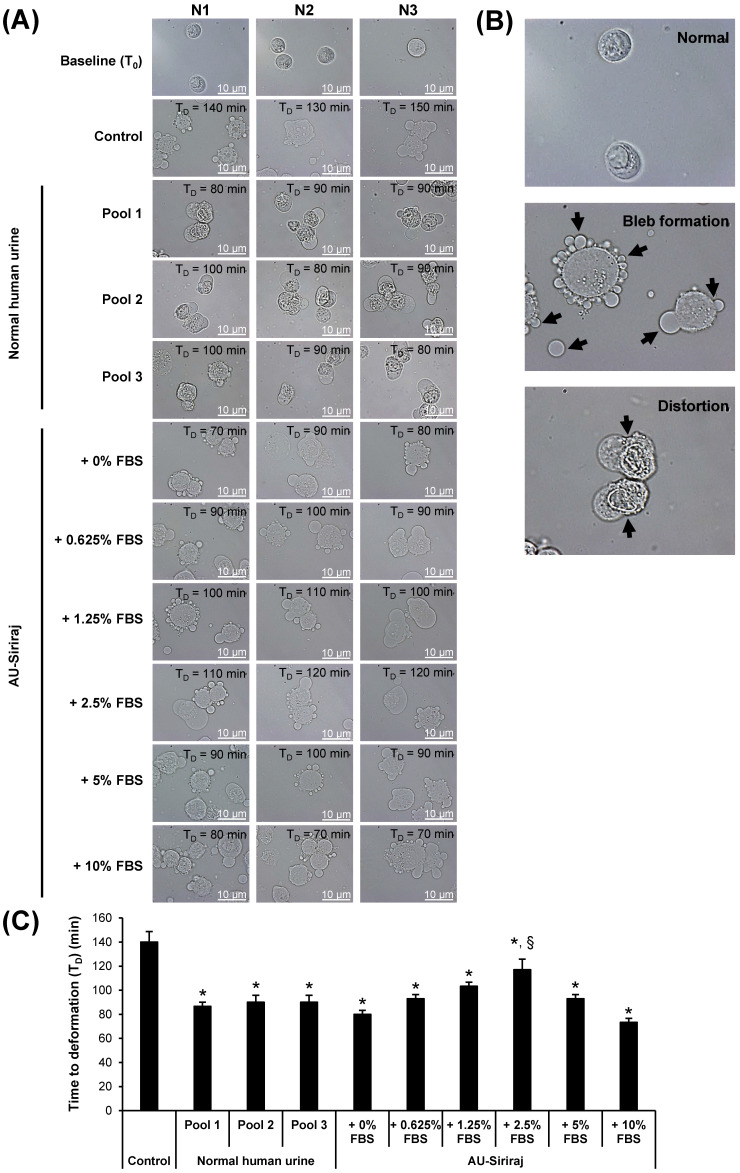
** Time to deformation (T_D_) of floating MDCK cells. (A):** The floating cells were incubated with the complete (serum-containing) medium (control), native (normal) human urine, or AU-Siriraj without or with FBS supplement. The cell morphology was monitored every 10 min under an inverted phase-contrast light microscope and the T_D_ was recorded. Original magnification = 1000× in all panels. Raw data obtained from all replicates of the cells in each group are provided in **Supplementary Figures S1-S10**. **(B):** Zoom-in images demonstrating bleb formation and cell distortion as compared with normal morphology of the floating cells. **(C):** Each bar represents mean ± SEM of the data obtained from three independent experiments. * = *p* < 0.05 shorter than control; § = *p* < 0.05 shorter than native urine and AU-Siriraj supplemented with other concentrations of FBS.

To evaluate the cell tolerance to the AU or native urine, the floating MDCK cells were incubated in the complete (serum-containing) medium, native urine, or AU-Siriraj with 0-10% FBS supplement. Cell morphology was monitored every 10 min under an inverted phase-contrast light microscope. The data showed that time to deformation (T_D_) significantly decreased for all cells exposed to the native urine or AU-Siriraj without/with FBS supplement compared with the control (**Figure 2 and Supplementary Figures S1-S10**). Comparing among the cells exposed to the native urine or AU, those incubated with AU-Siriraj + 2.5% FBS had the longest T_D_ (**Figure 2**).

**Figure 3:**
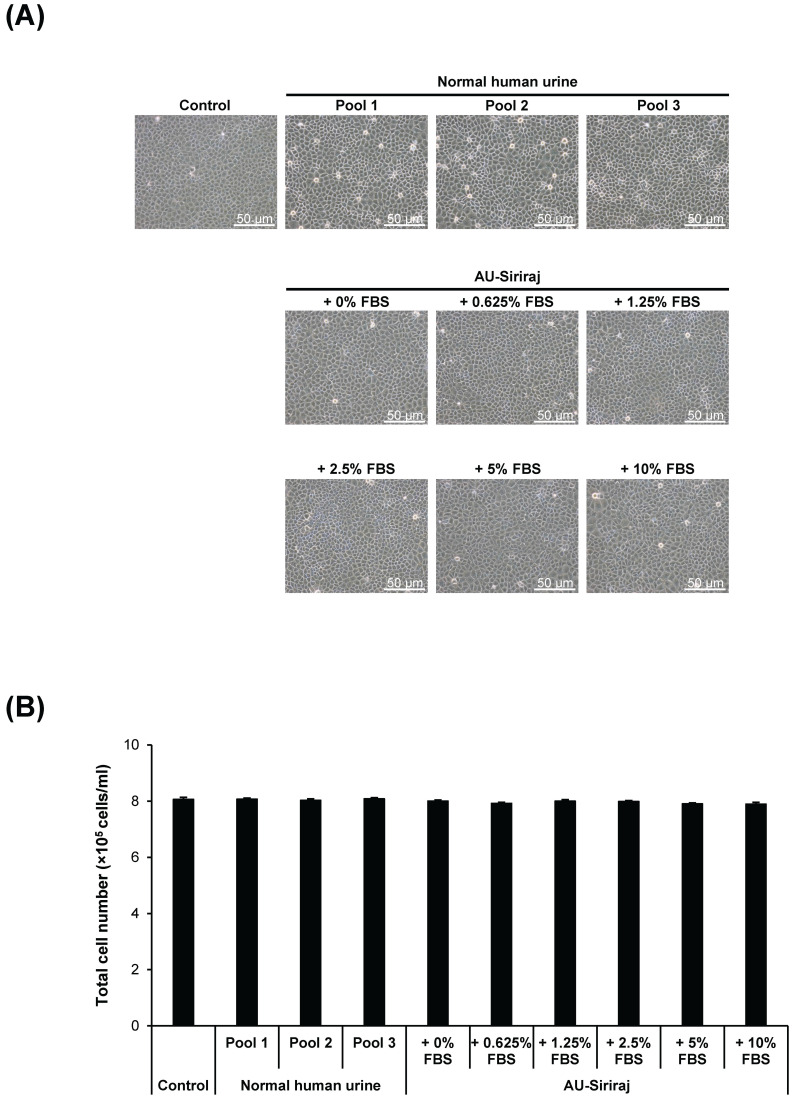
** Cell morphology and total number of the monolayered MDCK cells. (A):** The cell monolayers were incubated with the complete (serum-containing) medium (control), native (normal) human urine, or AU-Siriraj without or with FBS supplement. After 1-h incubation, the cell morphology was monitored under an inverted phase-contrast light microscope. Original magnification = 200× in all panels. **(B):** Total cell number was counted using hemacytometer. Each bar represents mean ± SEM of the data obtained from three independent experiments.

**Figure 4:**
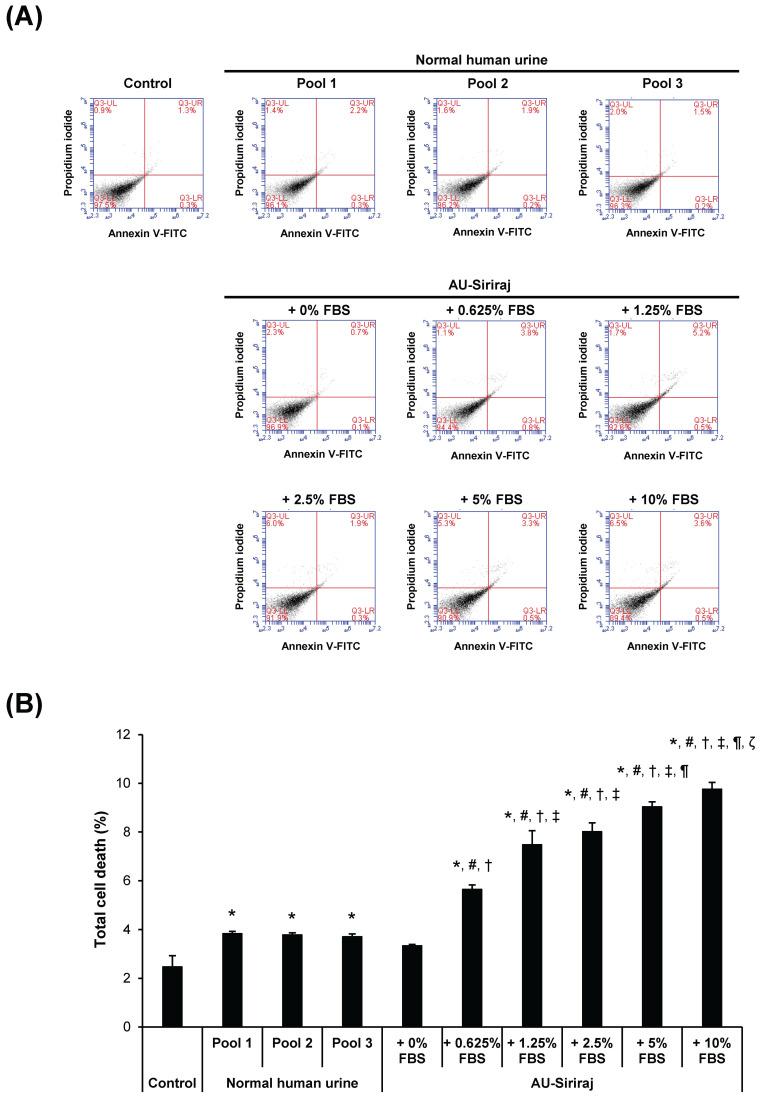
** Flow cytometric analysis of cell death in the monolayered MDCK cells. (A):** The cell monolayers were incubated with the complete (serum-containing) medium (control), native (normal) human urine, or AU-Siriraj without or with FBS supplement. After 1-h incubation, cell death was evaluated and quantified by flow cytometry with annexin V/propidium iodide co-staining. **(B):** Each bar represents mean ± SEM of the data obtained from three independent experiments. * = *p* < 0.05 greater than control; # = *p* < 0.05 greater than native urine; † = *p* < 0.05 greater than AU-Siriraj + 0% FBS; ‡ = *p* < 0.05 greater than AU-Siriraj + 0.625% FBS; ¶ = *p* < 0.05 greater than AU-Siriraj + 2.5% FBS; ζ = *p* < 0.05 greater than AU-Siriraj + 5% FBS.

To address the cytotoxic effects, cell morphology, total number and death were examined in the cell monolayers. After the monolayers completely formed, the cells were incubated with the complete (serum-containing) medium, native urine, or AU-Siriraj with 0-10% FBS supplement for 1 h. There were no obvious changes in cell morphology and total number observed in all groups (**Figure 3**). Flow cytometry using annexin V/propidium iodide co-staining was performed to evaluate early apoptosis, late apoptosis and necrosis in the cells. Comparing with the control, the cells had slightly greater percentage of total cell death when they were in the native urine, whereas AU-Siriraj without FBS supplement had no significant increase in the cell death (**Figure 4**). Supplementation of FBS significantly increased cell death in a dose-dependent manner (**Figure 4**).

**Figure 5:**
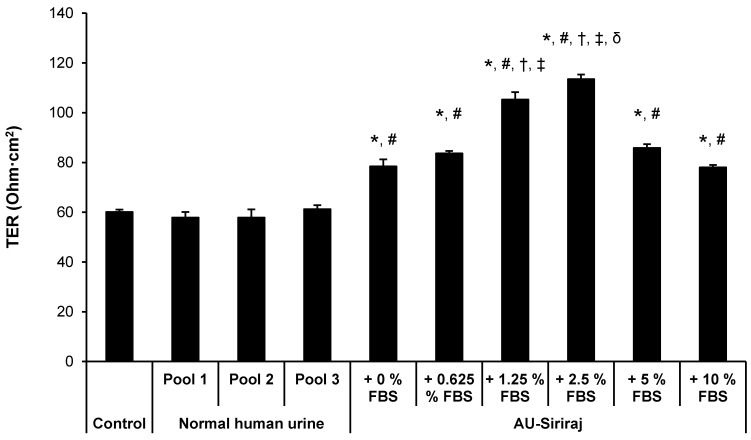
** Transepithelial electrical resistance (TER) of the polarized MDCK cells.** Polarization of the MDCK cells was induced using a Transwell system. After 60-h incubation with complete (serum-containing) medium, when TER was stabilized, the medium in the upper chamber was replaced with fresh complete (serum-containing) medium, native urine or AU-Siriraj without or with FBS supplement. After switching culture medium in the upper chamber for 24 h (or at 84-h post-culture), TER was measured. Each bar represents mean ± SEM of the data obtained from three independent experiments. * = *p* < 0.05 greater than control; # = *p* < 0.05 greater than native urine; † = *p* < 0.05 greater than AU-Siriraj + 0% FBS; ‡ = *p* < 0.05 greater than AU-Siriraj + 0.625% FBS; δ = *p* < 0.05 greater than AU-Siriraj + 1.25% FBS.

To analyze the cell integrity and polarization, TER was measured. After 60-h incubation, when TER was stabilized, the medium in upper chamber of each well of the Transwell plate was replaced with fresh complete (serum-containing) medium, native urine or AU-Siriraj without or with 0.625%, 1.25%, 2.5%, 5% or 10% FBS supplement. After switching culture medium in the upper chamber for 24 h (or at 84-h post-culture), TER was measured. The data showed that TER of the cells in the native urine was comparable to the control (**Figure 5**). Comparing with the control and native urine, all AU-Siriraj formulas without or with FBS supplement significantly increased the TER in the polarized cells. Among these, the cells incubated with AU-Siriraj + 2.5% FBS in the upper chamber of the Transwell had the highest TER, indicating that their fence function and polarization were most complete (**Figure 5**).

## 4. Discussion

The present study addressed whether supplementation of exogenous proteins from FBS could enhance cell biology and function *in vitro*. The AU formula used for such investigations was AU-Siriraj, which has been documented as one of the most physiologic AU formulas available to date 1. This physiologic AU formula has been widely used in several recent biomedical studies 2-12. It is suitable for secretome analysis and the study that avoids contaminations of exogenous proteins (i.e., evaluation of secretory endogenous cytokines/chemokines) 14-16. However, renal tubular cells are surrounded by the blood from capillary mesh at basolateral site, whereas their apical membranes are exposed to renal tubular fluid, which contains several various proteins filtrated from the plasma and secreted from the upstream cells 23-25. Therefore, it would be logical to formulate the most physiologic AU to simulate the native urine by supplementation of minimal amount of plasma/serum proteins.

For the native urine, diurnal variations are the important factors determining the urinary compositions 29, 30. The first or second morning urine is suitable for measurement of urinary protein concentration 29, 30. However, the first or second morning urine is not suitable for our present study because it can be associated with cytotoxicity 31, 32. In addition, 24-h urine is commonly used for precise measurement of urinary albumin/protein excretion as recommended by the Kidney Disease: Improving Global Outcomes (KDIGO) Clinical Practice Guideline 19. Nevertheless, the 24-h urine may have bacterial overgrowth and preservatives are usually added into the collecting container 33. By contrast, the recent data have shown that late afternoon urine is more appropriate and serves as the sample of choice for healthy children 34. In addition, the random afternoon urine from adults has been used for urinary proteome analysis and biomarker discovery 35-37. Moreover, early or late afternoon urine is more accurate to reflect 24-h urine osmolality and specific gravity as compared with morning, evening or overnight urine 36, 37. Along with practical points and recommendations for urinary proteome analysis by The Human Kidney and Urine Proteome Project (HKUPP) 29, 30, our study therefore used late afternoon midstream void to prevent the aforementioned confounding factors. Note that there were no significant differences of the effects from different native urine pools observed in all assays in this study (each of the three pools were collected from different groups of four individuals).

To screen for the optimal concentration of FBS that should be added into the AU-Siriraj formula, time to deformation (T_D_) of the floating MDCK cells was first evaluated. The T_D_ is one of the indicators for cell tolerance during treatment conditions 38, 39. The cell deformation can be determined when there is (are) cell shape change, shrinkage, membrane bleb formation, spiking, distortion, etc. 38, 39. It was not unexpected that both the native urine and AU formulas caused significant decrease in T_D_. In other words, the cells were less tolerant when they were exposed to native urine or AU compared with the complete (serum-containing) medium. Interestingly, supplementation of 2.5% FBS into the AU-Siriraj formula provided the longest T_D_ as compared with the other FBS concentrations. It's likely the optimal concentration of FBS that is compatible with the cell biology. Such improved tolerance might be the effect from proteins, osmolality, pH, chemicals, ions, or trace elements in the serum.

Exposure to an excess amount of urinary proteins (as in the case of macroalbuminuria or overt proteinuria) can induce cytotoxicity 40-42. Flow cytometry using annexin V/propidium iodide showed a slight increase in cell death by the native urine. This was not unexpected because the native urine contains proteins and also metabolites as well as waste products that might be responsible for such cytotoxic effects 40-42. This data was consistent with the T_D_ as aforementioned. It has been evidenced that cell deformability is associated with cytotoxicity and cell death, and the late stage of cell deformation can lead to a loss of the cell membrane and ultimately cellular disruption and death 43, 44. On the other hand, the protein-free AU-Siriraj formula did not induce the cell death, consistent with our previous study demonstrating that this AU formula is quite physiologic 1. However, supplementation of FBS significantly increased the cell death in a dose-dependent manner. Note that the total cell death remained < 10% for all conditions. In concordance, studies on different cell types have shown that the culture medium containing 10% FBS and 50% AU (using the protocol established by Griffith et al. 45) reduced cell viability and induced cytotoxicity in UROtsa cells after incubation for 24 h 46. Likewise, the prolonged incubation (for 7 days) with 10% FBS and 30% AU also induced the cytotoxicity 46. Again, the excess amount of proteins in the urine might be responsible for such cytotoxicity, consistent with the data reported previously for macroalbuminuria and overt proteinuria 40-42. It should be noted that FBS was derived from bovine, whereas MDCK cells were originated from canine. Difference in species may or may not generate a cross-reactivity. However, this effect (if any) is subtle as routine cell culture also uses FBS for MDCK cells without any problem of the cross-reactivity recognized.

Tight junction is important for the formation of apical intercellular junctional complex that governs fence function of the polarized cells. This junctional complex also plays roles in epithelial cell proliferation and differentiation 47, 48. Our present study measured TER, which represents the integrity of the tight junction and serves as an indicator of the fence function of the polarized cells. In concordance with our previous study 13, the AU-Siriraj without FBS supplement significantly increased the TER, indicating the more complete polarization as compared with the conventional polarized cell culture using complete medium for both upper and lower chambers. For the *in vivo* milieu, the apical membranes of renal tubular epithelial cells are exposed to renal tubular fluid (represented by AU in the upper chamber of the Transwell). On the other side, the basolateral membranes of renal tubular cells are exposed to blood and nutrients from capillary mesh (represented by the complete (serum-containing) medium in the lower chamber of the Transwell). Therefore, it was not surprising that using AU in the upper chamber could enhance the cell polarization 13. Finally, our data also demonstrated that supplementation of 2.5% FBS into the AU-Siriraj formula could enhance the TER to its highest level. These data indicate that 2.5% is the optimal concentration of FBS that is suitable for supplementation into the AU-Siriraj formula for cell biology and functional studies.

In summary, our findings indicate that AU-Siriraj supplemented with 2.5% FBS is superior to the native urine and AU-Siriraj without FBS supplementation in terms of the cell tolerance (determined by T_D_) and cell polarization (determined by TER). Higher concentrations of the supplemental FBS do not improve the cell tolerance and polarization, and on the other hand, induce greater cell death. Therefore, supplementation of 2.5% FBS into the AU-Siriraj formula is highly recommended for the *in vitro* study of cell biology and physiology. However, the protein-free AU-Siriraj is still useful, particularly for secretome analysis and the study of secretory endogenous proteins that cannot compromise the interference from exogenous proteins.

## Supplementary Material

Supplementary figures.
