# Supplementary material for: Optimization of artificial urine formula for in vitro cellular study compared with native urine
Source: Int J Med Sci. 2021 Jul 23;18(14):3271–9. doi: 10.7150/ijms.61720 (PMC8364465; doi:10.7150/ijms.61720)
Supplement: Supplementary file 1 — Supplementary figures. [file ijmsv18p3271s1.pdf]

SUPPLEMENTARY MATERIALS

Optimization of artificial urine formula for *in vitro* cellular study compared with native urine

*Kanyarat Sueksakit and Visith Thongboonkerd**

* E-mail: thongboonkerd@dr.com (or) vthongbo@yahoo.com

Supplementary Figure S1: Serial monitoring of time to deformation (T_D) of floating MDCK cells incubated with the complete (serum-containing) medium (control). **(A)-(C):** Raw data of all triplicates are shown. The T_D of each replicate (as shown in **Figure 2**) (when cells had bleb formation or shape distortion) is highlighted with the red dashed box. Original magnification = 400X for all panels.

Supplementary Figure S2: Serial monitoring of time to deformation (T_D) of floating MDCK cells incubated with the native (normal) human urine Pool 1. **(A)-(C):** Raw data of all triplicates are shown. The T_D of each replicate (as shown in **Figure 2**) (when cells had bleb formation or shape distortion) is highlighted with the red dashed box. Original magnification = 400X for all panels.

Supplementary Figure S3: Serial monitoring of time to deformation (T_D) of floating MDCK cells incubated with the native (normal) human urine Pool 2. **(A)-(C):** Raw data of all triplicates are shown. The T_D of each replicate (as shown in **Figure 2**) (when cells had bleb formation or shape distortion) is highlighted with the red dashed box. Original magnification = 400X for all panels.

Supplementary Figure S4: Serial monitoring of time to deformation (T_D) of floating MDCK cells incubated with the native (normal) human urine Pool 3. **(A)-(C):** Raw data of all triplicates are shown. The T_D of each replicate (as shown in **Figure 2**) (when cells had bleb formation or shape distortion) is highlighted with the red dashed box. Original magnification = 400X for all panels.

Supplementary Figure S5: Serial monitoring of time to deformation (T_D) of floating MDCK cells incubated with the AU-Siriraj without FBS supplement. **(A)-(C):** Raw data of all triplicates are shown. The T_D of each replicate (as shown in **Figure 2**) (when cells had bleb formation or shape distortion) is highlighted with the red dashed box. Original magnification = 400X for all panels.

Supplementary Figure S6: Serial monitoring of time to deformation (T_D) of floating MDCK cells incubated with the AU-Siriraj supplemented with 0.625% FBS. **(A)-(C):** Raw data of all triplicates are shown. The T_D of each replicate (as shown in **Figure 2**) (when cells had bleb formation or shape distortion) is highlighted with the red dashed box. Original magnification = 400X for all panels.

Supplementary Figure S7: Serial monitoring of time to deformation (T_D) of floating MDCK cells incubated with the AU-Siriraj supplemented with 1.25% FBS. **(A)-(C):** Raw data of all triplicates are shown. The T_D of each replicate (as shown in **Figure 2**) (when cells had bleb formation or shape distortion) is highlighted with the red dashed box. Original magnification = 400X for all panels.

Supplementary Figure S8: Serial monitoring of time to deformation (T_D) of floating MDCK cells incubated with the AU-Siriraj supplemented with 2.5% FBS. **(A)-(C):** Raw data of all triplicates are shown. The T_D of each replicate (as shown in **Figure 2**) (when cells had bleb formation or shape distortion) is highlighted with the red dashed box. Original magnification = 400X for all panels.

Supplementary Figure S9: Serial monitoring of time to deformation (T_D) of floating MDCK cells incubated with the AU-Siriraj supplemented with 5% FBS. **(A)-(C):** Raw data of all triplicates are shown. The T_D of each replicate (as shown in **Figure 2**) (when cells had bleb formation or shape distortion) is highlighted with the red dashed box. Original magnification = 400X for all panels.

Supplementary Figure S10: Serial monitoring of time to deformation (T_D) of floating MDCK cells incubated with the AU-Siriraj supplemented with 10% FBS. **(A)-(C):** Raw data of all triplicates are shown. The T_D of each replicate (as shown in **Figure 2**) (when cells had bleb formation or shape distortion) is highlighted with the red dashed box. Original magnification = 400X for all panels.

Figure S1

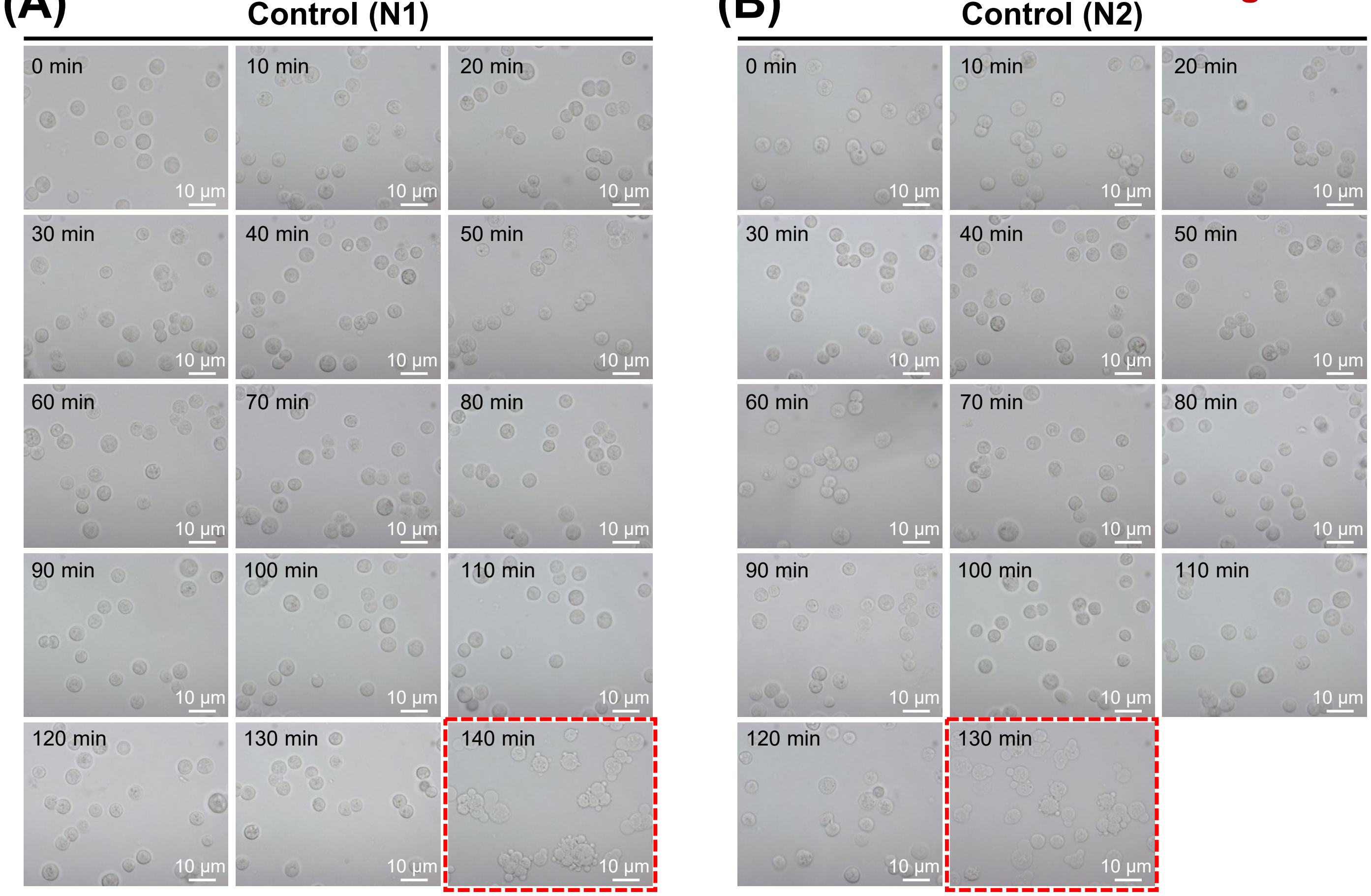

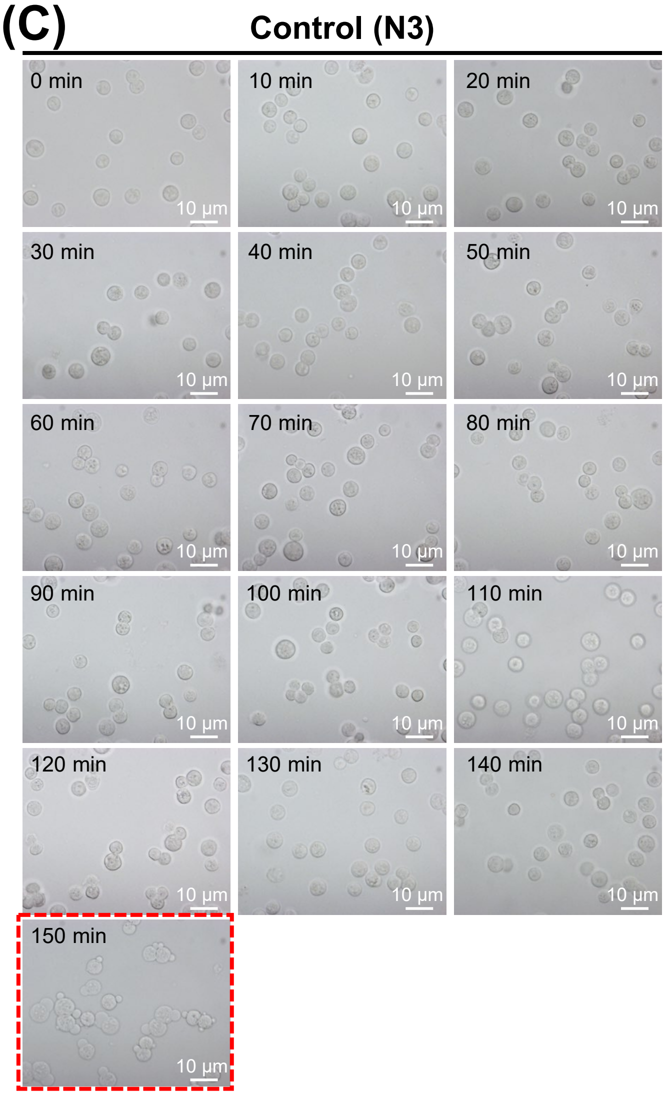

Supplementary Figure S1: Serial monitoring of time to deformation (T_D) of floating MDCK cells incubated with the complete (serum-containing) medium (control). **(A)-(C):** Raw data of all triplicates are shown. The T_D of each replicate (as shown in **Figure 2**) (when cells had bleb formation or shape distortion) is highlighted with the red dashed box. Original magnification = 400X for all panels.

Figure S2

(A)

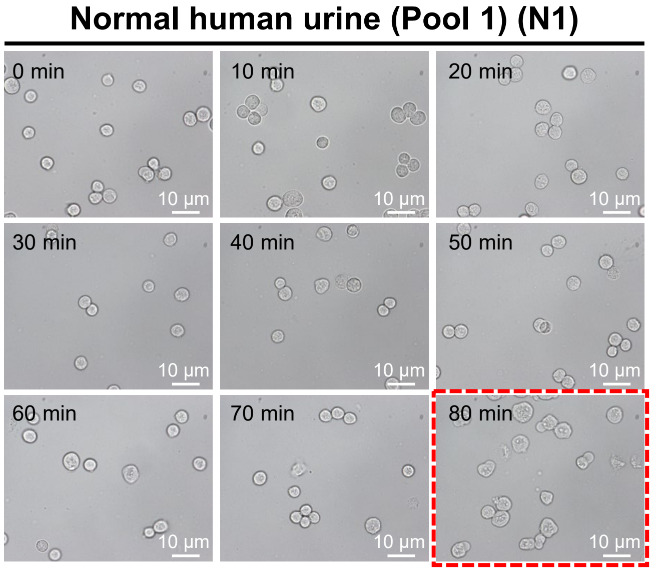

(B)

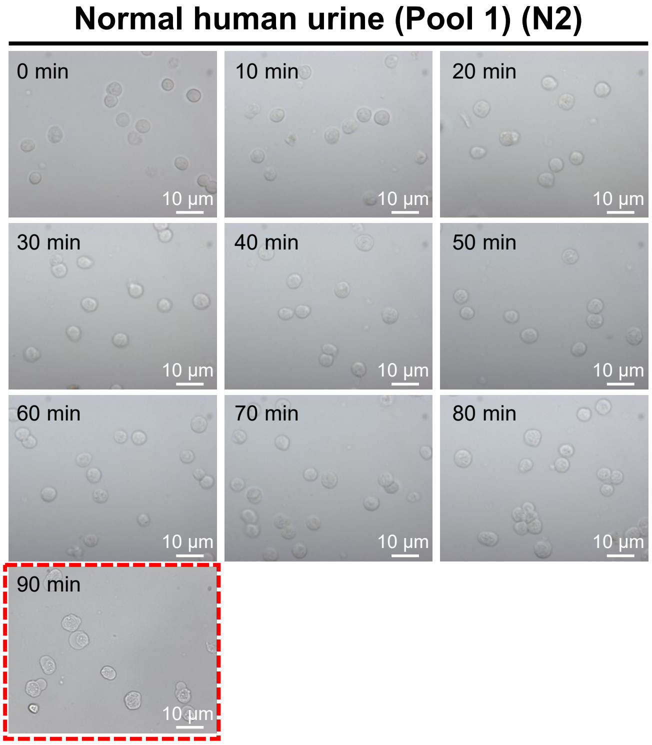

(C)

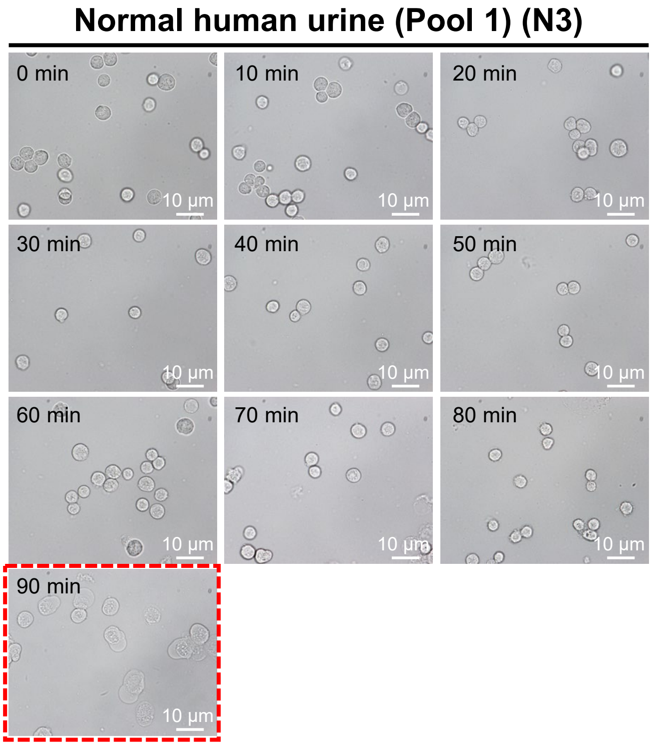

Supplementary Figure S2: Serial monitoring of time to deformation (T_D) of floating MDCK cells incubated with the native (normal) human urine Pool 1. **(A)-(C):** Raw data of all triplicates are shown. The T_D of each replicate (as shown in **Figure 2**) (when cells had bleb formation or shape distortion) is highlighted with the red dashed box. Original magnification = 400X for all panels.

Figure S3

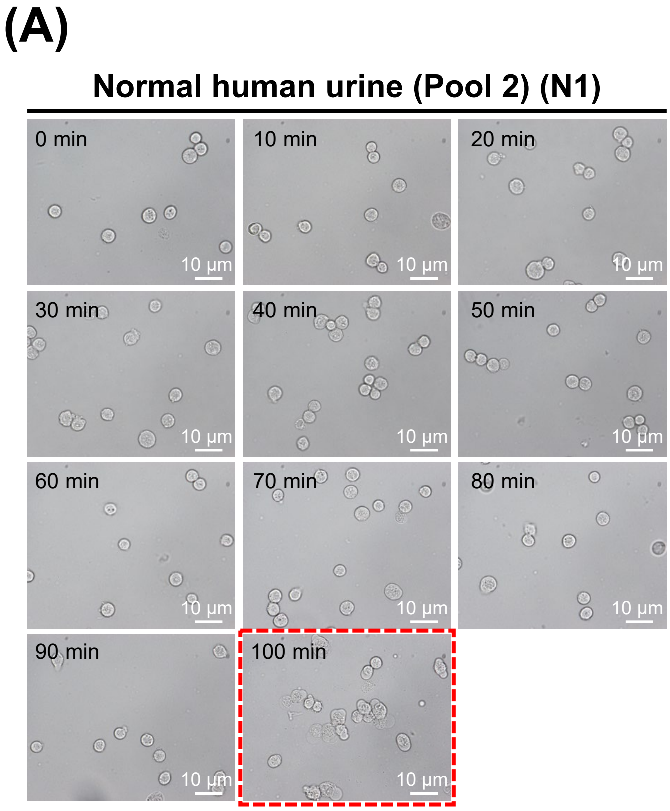

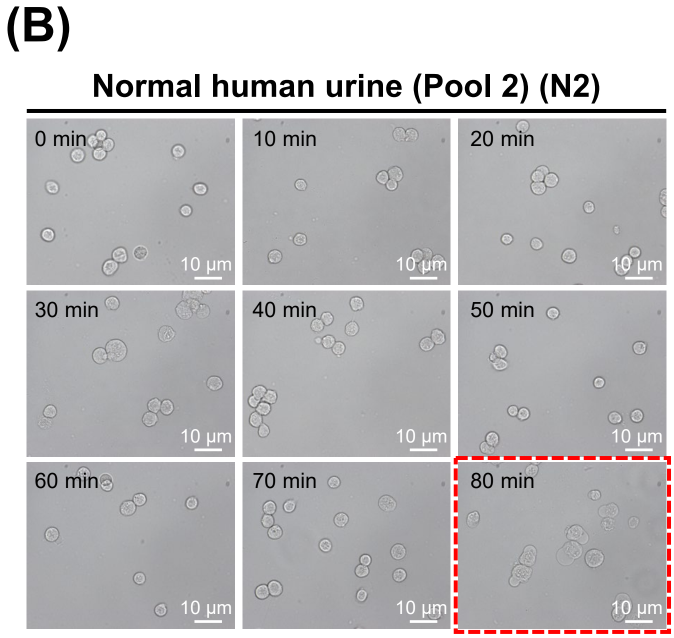

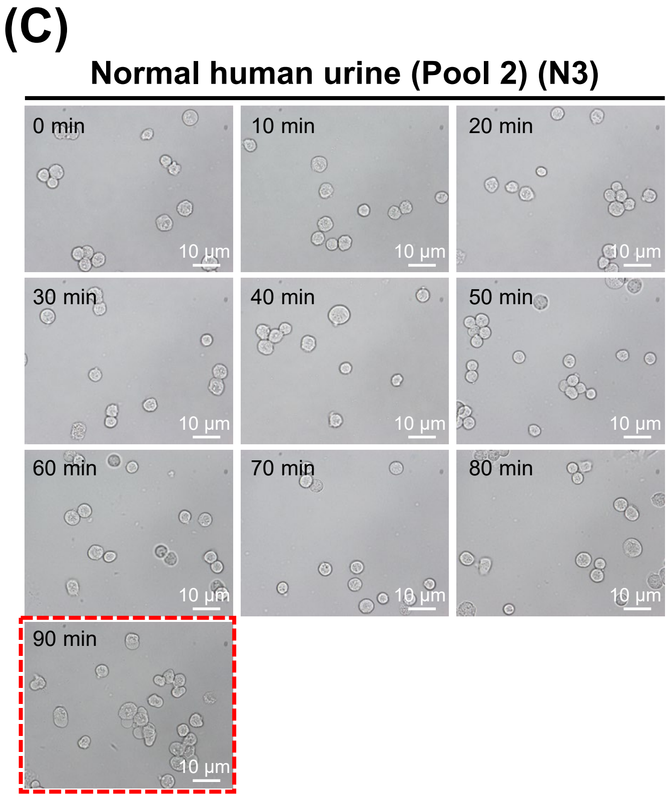

Supplementary Figure S3: Serial monitoring of time to deformation (T_D) of floating MDCK cells incubated with the native (normal) human urine Pool 2. **(A)-(C):** Raw data of all triplicates are shown. The T_D of each replicate (as shown in **Figure 2**) (when cells had bleb formation or shape distortion) is highlighted with the red dashed box. Original magnification = 400X for all panels.

(A)

Normal human urine (Pool 3) (N1)

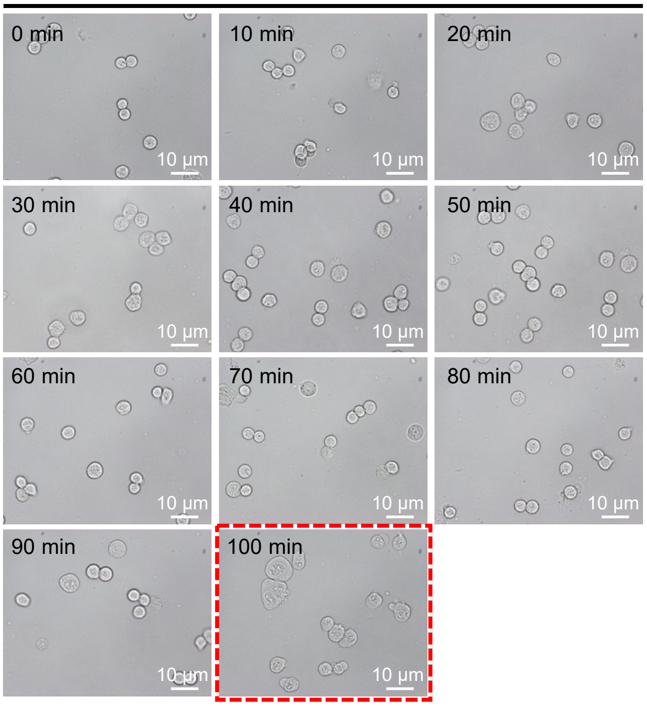

(B)

Normal human urine (Pool 3) (N2)

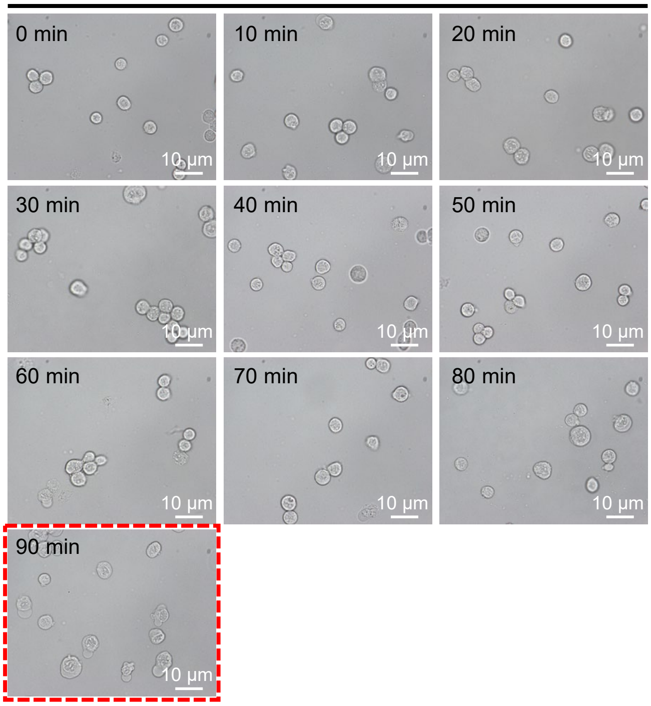

(C)

Normal human urine (Pool 3) (N3)

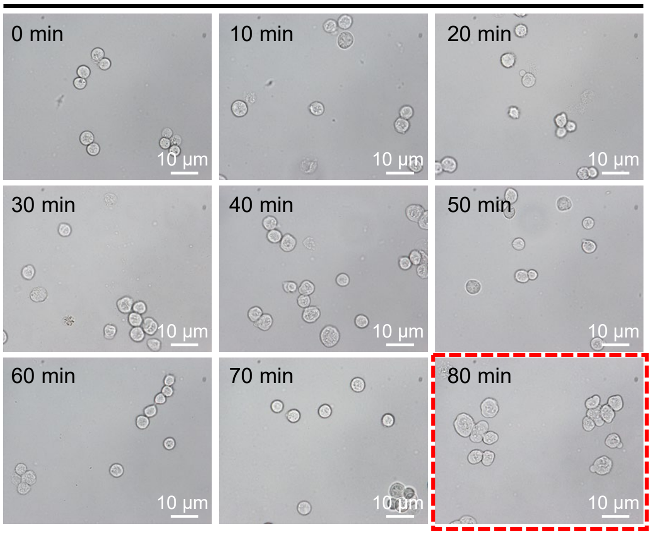

Supplementary Figure S4: Serial monitoring of time to deformation (T_D) of floating MDCK cells incubated with the native (normal) human urine Pool 3. **(A)-(C):** Raw data of all triplicates are shown. The T_D of each replicate (as shown in **Figure 2**) (when cells had bleb formation or shape distortion) is highlighted with the red dashed box. Original magnification = 400X for all panels.

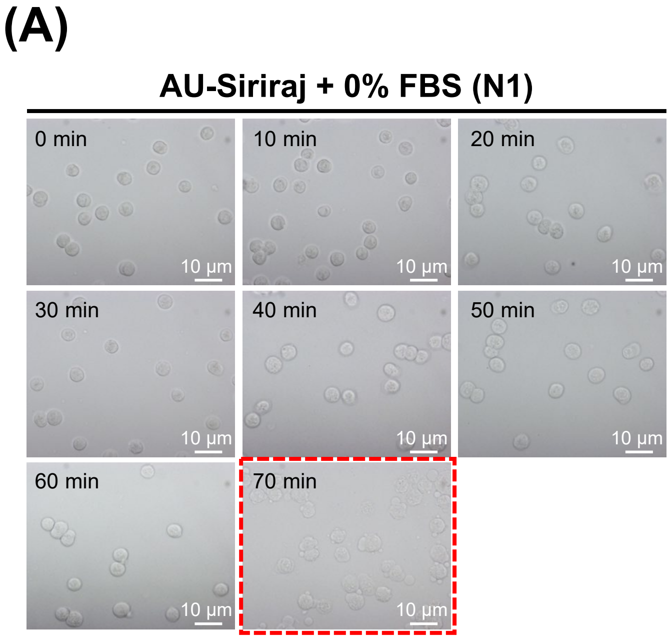

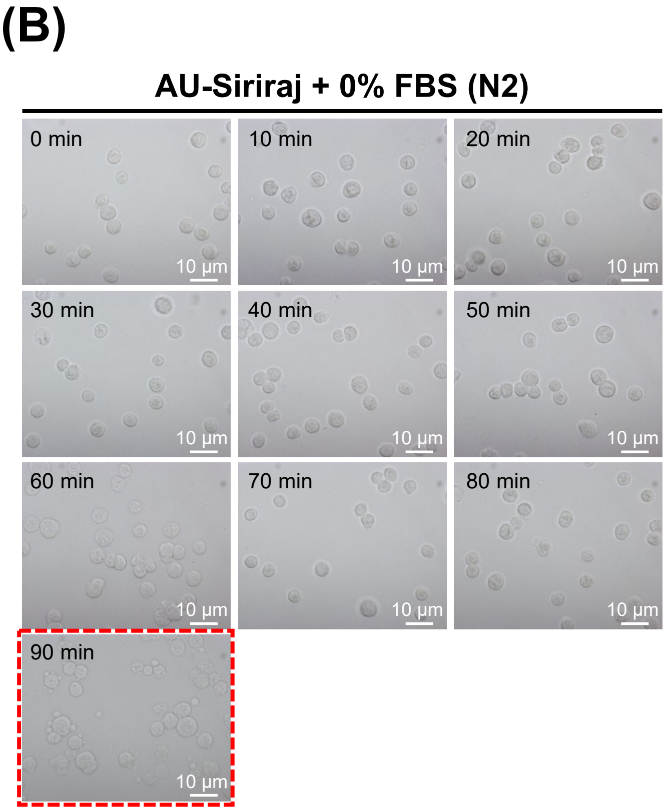

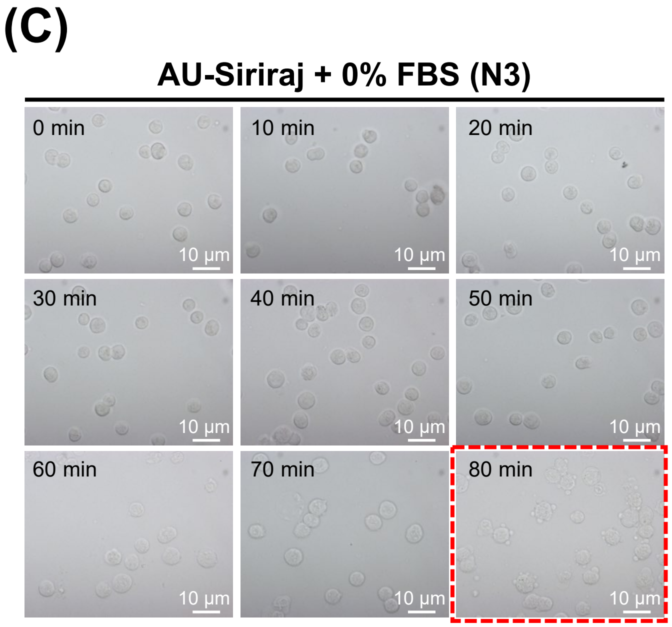

Supplementary Figure S5: Serial monitoring of time to deformation (T_D) of floating MDCK cells incubated with the AU-Siriraj without FBS supplement. **(A)-(C):** Raw data of all triplicates are shown. The T_D of each replicate (as shown in **Figure 2**) (when cells had bleb formation or shape distortion) is highlighted with the red dashed box. Original magnification = 400X for all panels.

Figure S6

(A)

AU-Siriraj + 0.625% FBS (N1)

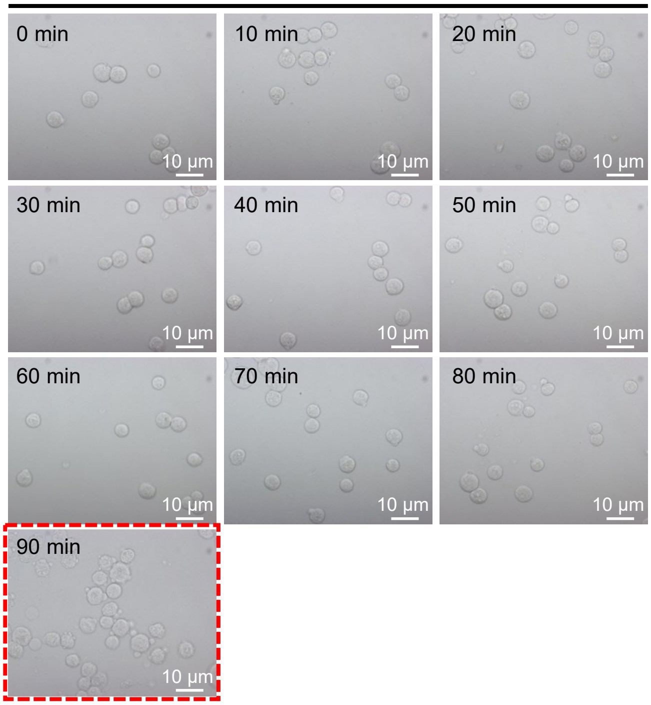

(B)

AU-Siriraj + 0.625% FBS (N2)

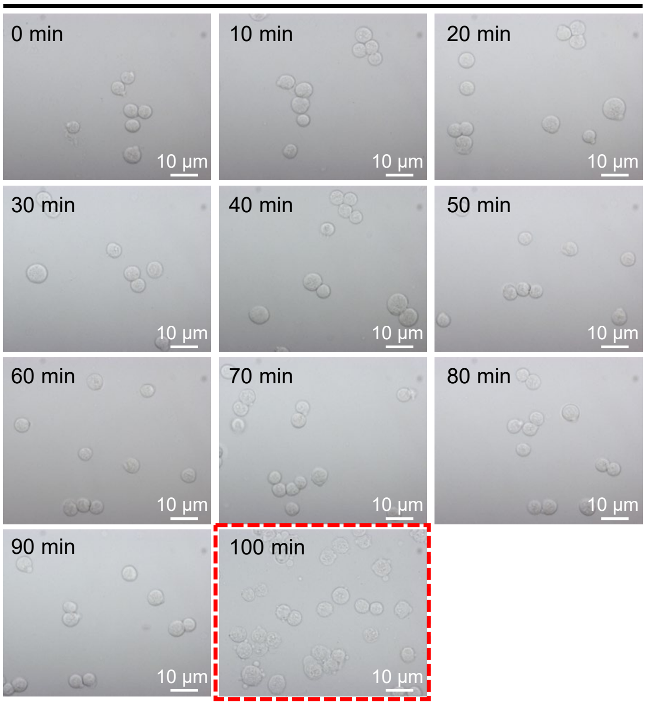

(C)

AU-Siriraj + 0.625% FBS (N3)

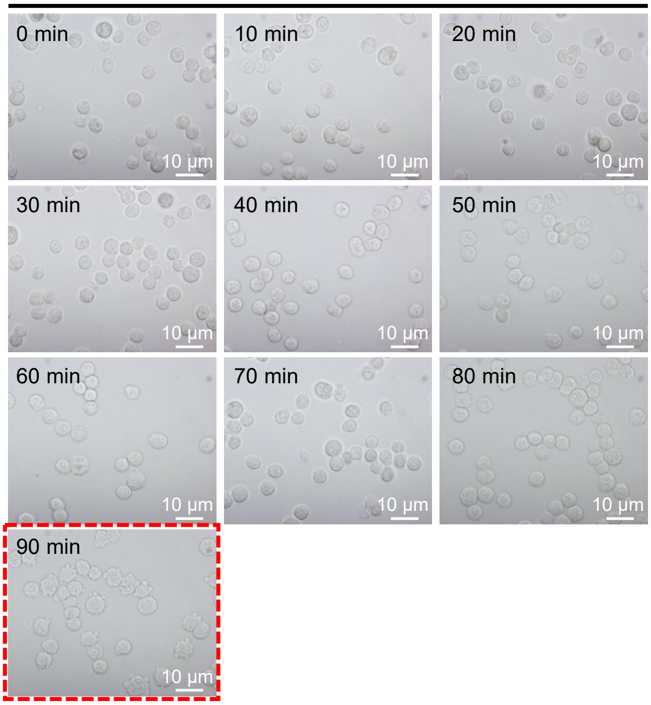

Supplementary Figure S6: Serial monitoring of time to deformation (T_D) of floating MDCK cells incubated with the AU-Siriraj supplemented with 0.625% FBS. **(A)-(C):** Raw data of all triplicates are shown. The T_D of each replicate (as shown in **Figure 2**) (when cells had bleb formation or shape distortion) is highlighted with the red dashed box. Original magnification = 400X for all panels.

Figure S7

(A)

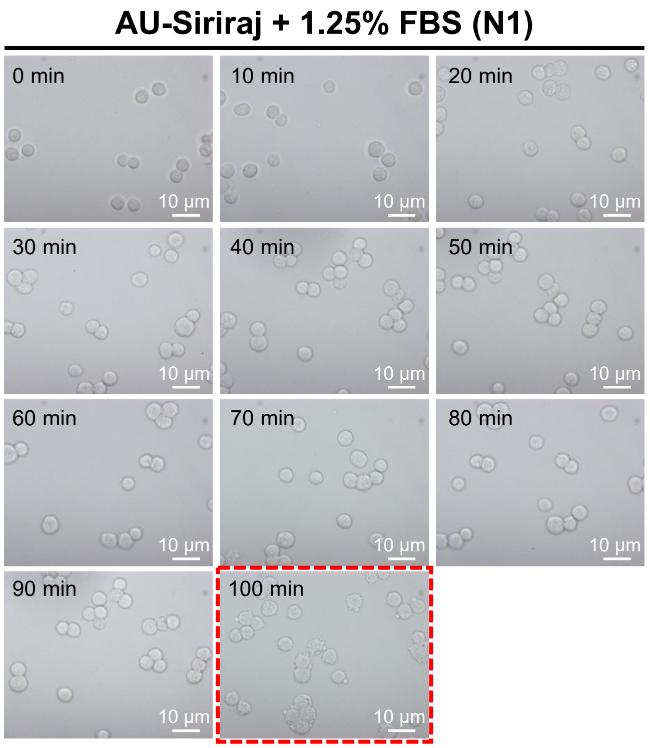

(B)

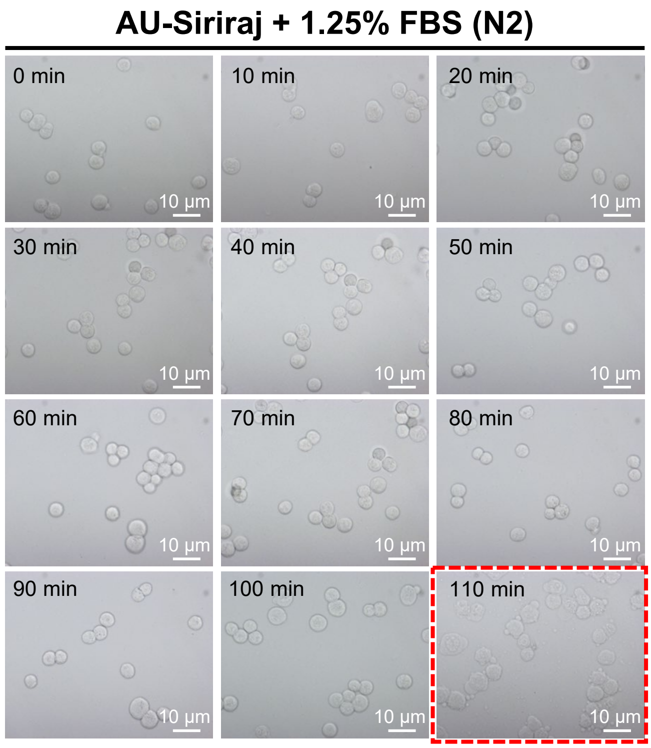

(C)

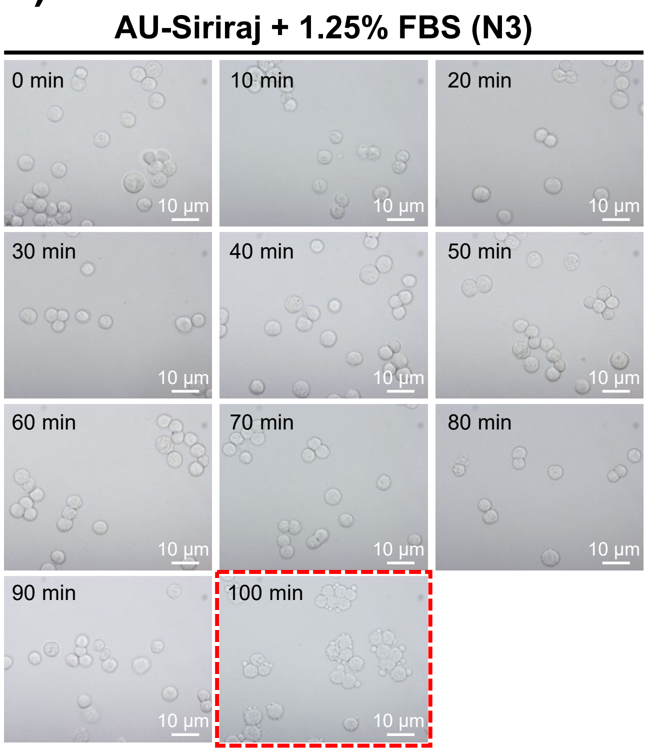

Supplementary Figure S7: Serial monitoring of time to deformation (T_D) of floating MDCK cells incubated with the AU-Siriraj supplemented with 1.25% FBS. **(A)-(C):** Raw data of all triplicates are shown. The T_D of each replicate (as shown in **Figure 2**) (when cells had bleb formation or shape distortion) is highlighted with the red dashed box. Original magnification = 400X for all panels.

Figure S8

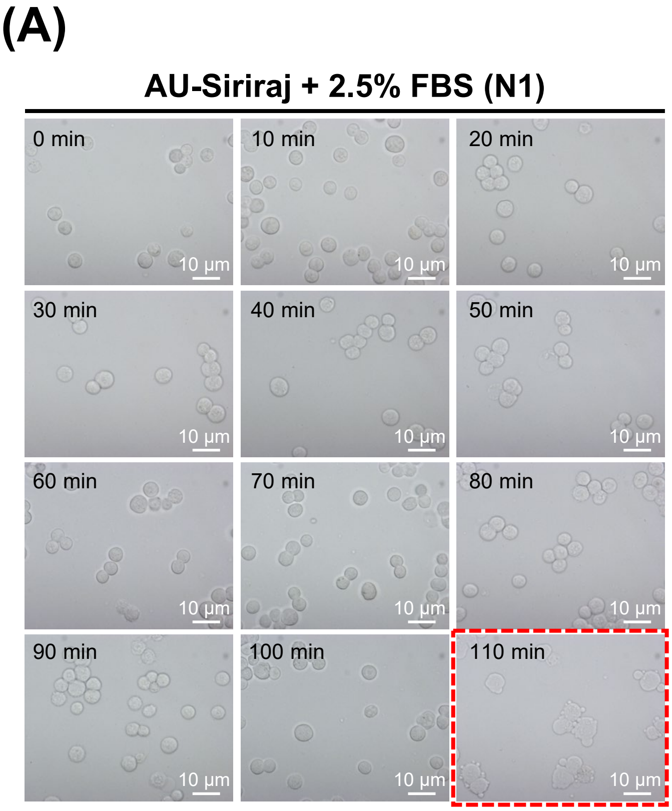

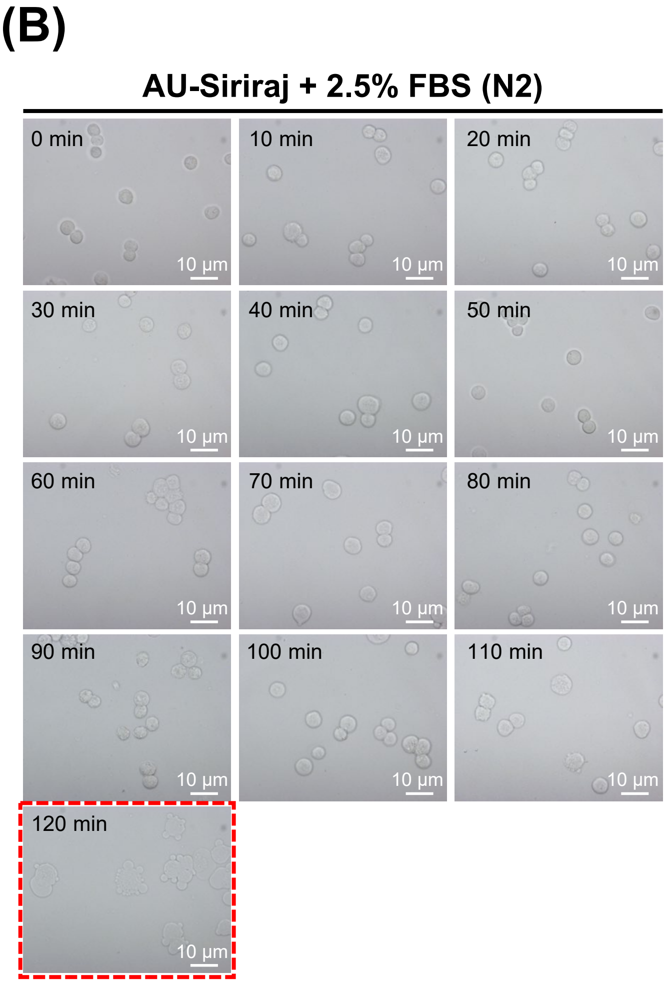

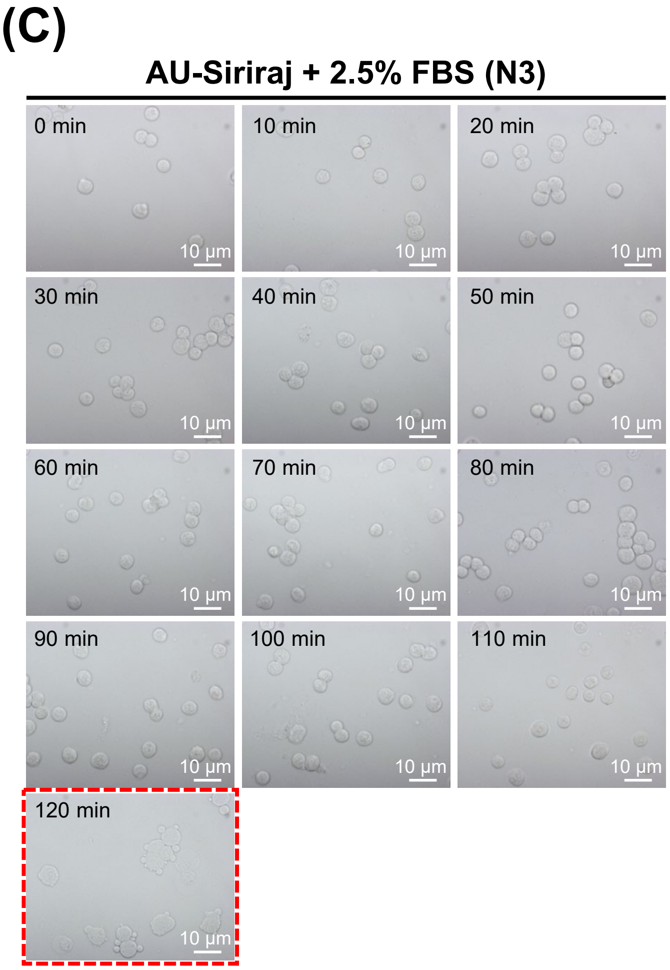

Supplementary Figure S8: Serial monitoring of time to deformation (T_D) of floating MDCK cells incubated with the AU-Siriraj supplemented with 2.5% FBS. **(A)-(C):** Raw data of all triplicates are shown. The T_D of each replicate (as shown in **Figure 2**) (when cells had bleb formation or shape distortion) is highlighted with the red dashed box. Original magnification = 400X for all panels.

Figure S9

(A)

AU-Siriraj + 5% FBS (N1)

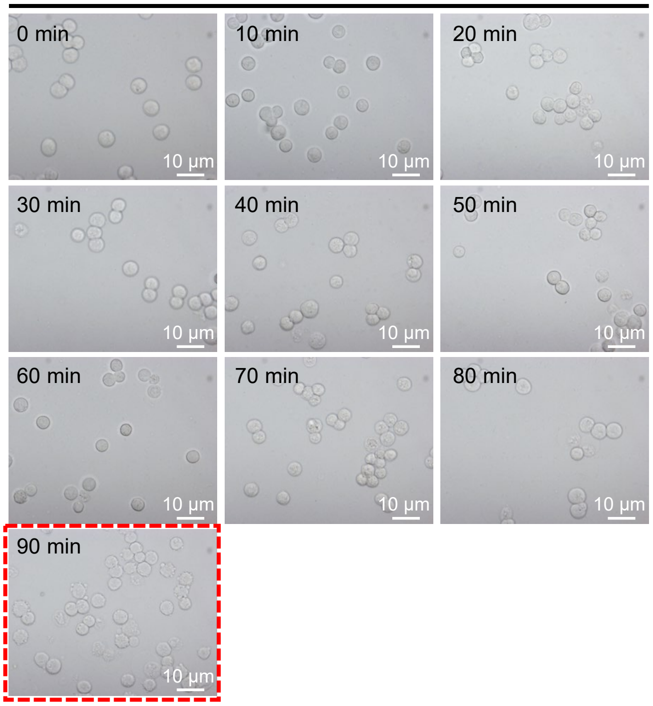

(B)

AU-Siriraj + 5% FBS (N2)

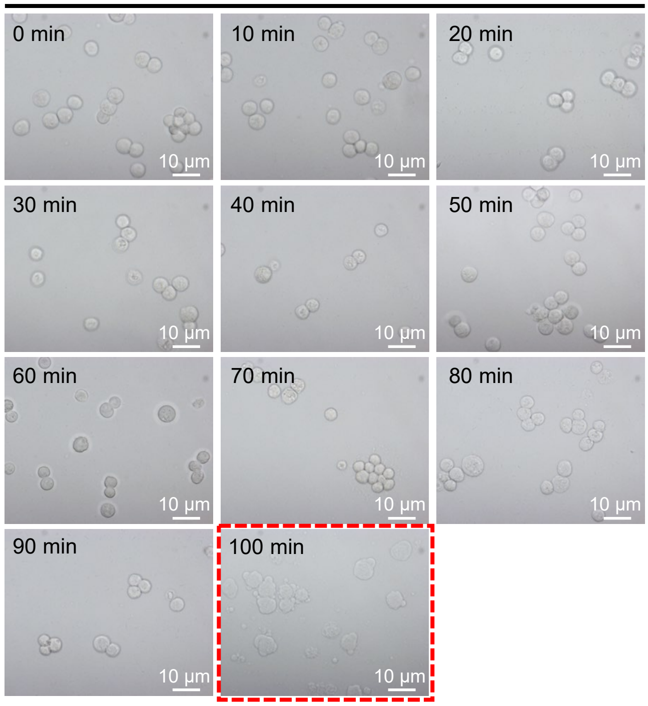

(C)

AU-Siriraj + 5% FBS (N3)

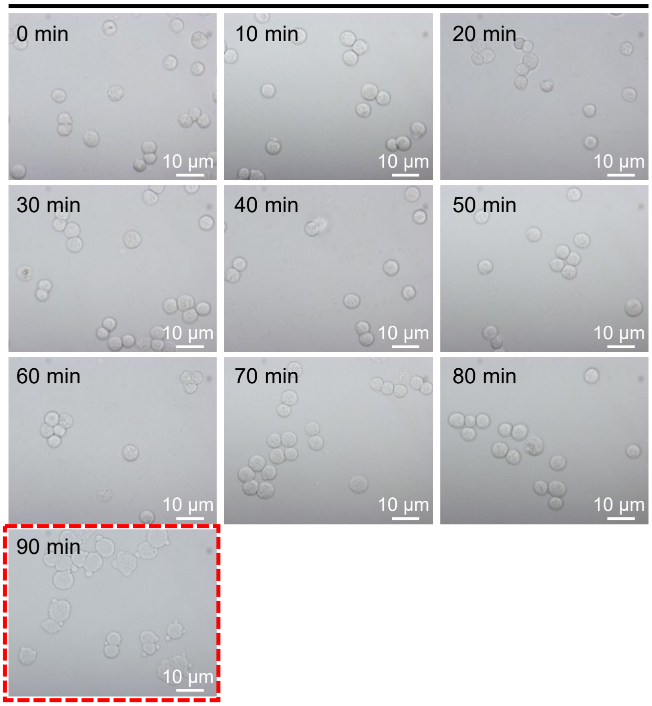

Supplementary Figure S9: Serial monitoring of time to deformation (T_D) of floating MDCK cells incubated with the AU-Siriraj supplemented with 5% FBS. **(A)-(C):** Raw data of all triplicates are shown. The T_D of each replicate (as shown in **Figure 2**) (when cells had bleb formation or shape distortion) is highlighted with the red dashed box. Original magnification = 400X for all panels.

(A)

AU-Siriraj + 10% FBS (N1)

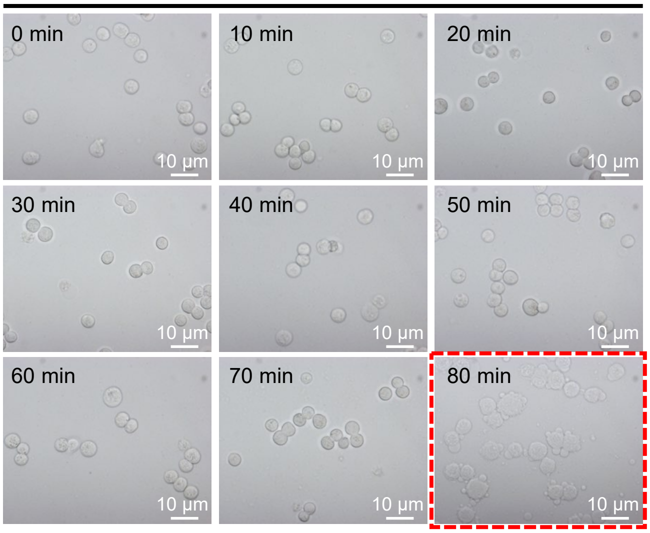

(B)

AU-Siriraj + 10% FBS (N2)

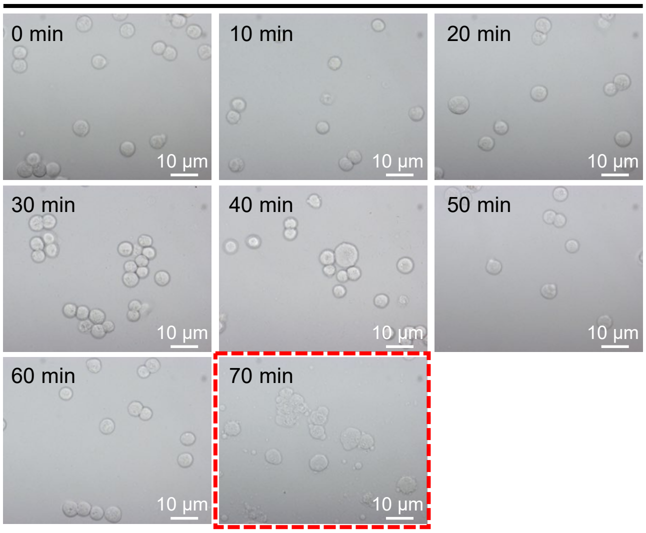

(C)

AU-Siriraj + 10% FBS (N3)

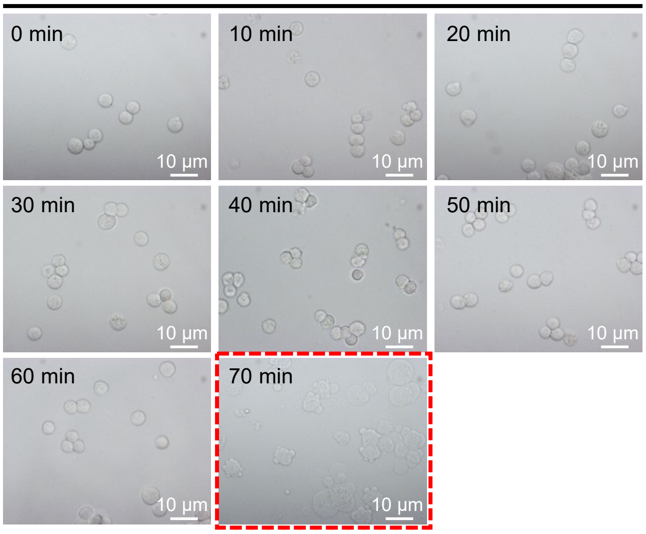

Supplementary Figure S10: Serial monitoring of time to deformation (T_D) of floating MDCK cells incubated with the AU-Siriraj supplemented with 10% FBS. **(A)-(C):** Raw data of all triplicates are shown. The T_D of each replicate (as shown in **Figure 2**) (when cells had bleb formation or shape distortion) is highlighted with the red dashed box. Original magnification = 400X for all panels.
